# Time-Series Growth Prediction Model Based on U-Net and Machine Learning in *Arabidopsis*

**DOI:** 10.3389/fpls.2021.721512

**Published:** 2021-11-11

**Authors:** Sungyul Chang, Unseok Lee, Min Jeong Hong, Yeong Deuk Jo, Jin-Baek Kim

**Affiliations:** ^1^Radiation Breeding Research Team, Advanced Radiation Technology Institute (ARTI), Korea Atomic Energy Research Institute (KAERI), Jeongeup-si, South Korea; ^2^Smart Farm Research Center, Korea Institute of Science and Technology (KIST), Gangneung-si, South Korea

**Keywords:** time series analysis, phenomics, high-throughput phenotyping (HTP), deep learning DL), growth modeling, plant biomass, *Arabidopsis thaliana*

## Abstract

Yield prediction for crops is essential information for food security. A high-throughput phenotyping platform (HTPP) generates the data of the complete life cycle of a plant. However, the data are rarely used for yield prediction because of the lack of quality image analysis methods, yield data associated with HTPP, and the time-series analysis method for yield prediction. To overcome limitations, this study employed multiple deep learning (DL) networks to extract high-quality HTTP data, establish an association between HTTP data and the yield performance of crops, and select essential time intervals using machine learning (ML). The images of *Arabidopsis* were taken 12 times under environmentally controlled HTPP over 23 days after sowing (DAS). First, the features from images were extracted using DL network U-Net with SE-ResXt101 encoder and divided into early (15–21 DAS) and late (∼21–23 DAS) pre-flowering developmental stages using the physiological characteristics of the *Arabidopsis* plant. Second, the late pre-flowering stage at 23 DAS can be predicted using the ML algorithm XGBoost, based only on a portion of the early pre-flowering stage (17–21 DAS). This was confirmed using an additional biological experiment (*P* < 0.01). Finally, the projected area (PA) was estimated into fresh weight (FW), and the correlation coefficient between FW and predicted FW was calculated as 0.85. This was the first study that analyzed time-series data to predict the FW of related but different developmental stages and predict the PA. The results of this study were informative and enabled the understanding of the FW of *Arabidopsis* or yield of leafy plants and total biomass consumed in vertical farming. Moreover, this study highlighted the reduction of time-series data for examining interesting traits and future application of time-series analysis in various HTPPs.

## Introduction

Food insecurity has threatened the survival of many people because of the desertification of arable land, global climate changes, population increase (Godfray et al., 2010), and spread of infectious disease worldwide (Laborde et al., 2020). To combat food insecurity, agricultural production approaches have not been revamped, wherein “digital agriculture” was proposed to overcome these challenges (Redmond Ramin et al., 2018b). Multiple studies examined this concept about agricultural production (Waltz, 2017). Regardless of the food production method for growing field crops in indoor conditions, multiple challenges limit the implementation of this idea for the current agricultural production. The successful transformation requires digital plant phenotyping data and analysis tools (Granier and Vile, 2014). Determining plant performance in various situations requires various quantitative data to compare and make a decision (Großkinsky et al., 2015). Therefore, a description of the performance of a plant at a given time is important for the transformation of digital agriculture (Chawade et al., 2019).

Plant phenotype includes multiple aspects of plant science and its definitions vary in different plant science-related fields (Tardieu et al., 2017). Automated high-throughput phenotyping platform (HTPP) generates high-quality data (Lee et al., 2018) from multiple sensors (Fahlgren et al., 2015) and yields the complete life cycle of a plant (van Es et al., 2019). Moreover, rich phenotype data, based on time series generated from a single plant captured by HTPP, can provide insights into traits of interest. HTPP-generated data are used to investigate the salinity stress response in multiple rice cultivars and these data revealed that candidate genes can be resistant to salt-related stress (Al-Tamimi et al., 2016). However, many studies use only a small fraction of phenotype data for a fixed time point (Al-Tamimi et al., 2016; Chen et al., 2018) to associate phenotype data with interesting traits. This is primarily attributed to multiple plant scientists selecting measurement time that discriminates with notable traits in plant-related populations. Moreover, time-series analysis methods based on statistical models do not provide satisfying results (Boken, 2000). Recently, yield prediction for crop plants using machine learning (ML) algorithms from satellite or drone images provided high accuracies (Khaki et al., 2020). In these studies, the frequency of image acquisition is broad (days) and small changes over narrow (hours) time intervals are difficult to identify. Moreover, for determining phenotype changes over the plant life cycle, the examination of both narrow and broad time intervals is important (Tardieu et al., 2017). Novel time points with ML tools are essential because examining interesting traits from prior knowledge provides limited information on traits. The analysis and prediction of leaf area using time-series data at specific growth stages can establish prediction models for the growth pattern of a plant and essential time points. This study employed extreme gradient boost (XGBoost) for multiple time steps of forecast models. XGBoost, known as multiple additive regression trees, adds multiple decision trees to achieve the best outcome. XGBoost was used to analyze various classification and regression data not provided (Ji et al., 2019). It used multiple steps to make ensemble models for multiple time-step forecasts (Galicia et al., 2019). The additional benefits of using the ensemble models were the robustness and simplicity of modeling while forecasting (Dineva et al., 2020).

**FIGURE 1:**
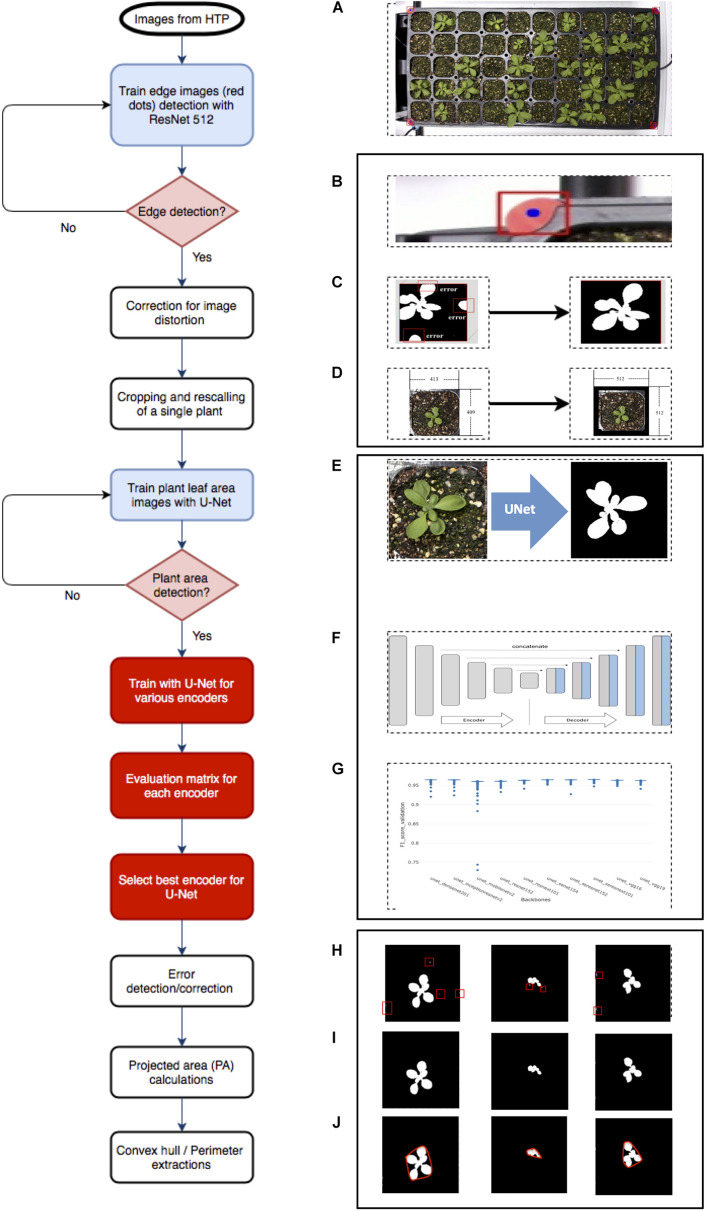
Overview of *Arabidopsis* image analysis pipeline. There are three steps for image analysis. The first step is the preprocessing of raw images by **(A)** the acquisition of the raw image, **(B)** detecting reference point (red dot), **(C)** correcting images with the red dot, **(D)** cropping into single plant images, and rescaling. The second step is to train the **(E)** U-Net with various encoders and selecting encoders for the best result using the U-Net structure, **(F)** including encoder section, training network with various encoders, such as SE-RENext101, **(G)** comparing results from various encoders. The last step is the post-processing of images and exporting data using **(H)** error detection with a conditional random feature, **(I)** extract PA, and convex hull area, **(J)** perimeter.

Machine learning-based analysis improved the extraction of projected area (PA) related to multiple agronomical traits. Many studies on the growth pattern of a plant are destructive, i.e., they harvest the plant to measure its weight. This method is labor-intensive, producing only a few time point measurements. HTPP gathers images related to plant weight in the PA with a high-frequency rate within a day. Moreover, the PA extracted from HTPP in this study showed a high correlation between images and biomass or photosynthetic capacity (Salas Fernandez et al., 2017). Similarly, multiple agricultural traits are directly or indirectly associated with PA (Yang et al., 2013; Araus and Cairns, 2014). Accurately extracting PA from the image of a plant is difficult because multiple size leaf areas are connected with thin branches in an overlapping manner (Lee et al., 2018). Previously, studies separated the plant area from background images, and the reported evaluation matrix shows that the accuracies of the segmentation of plants heavily depend on a specific dataset (Jiang and Li, 2020). ML algorithms, such as random forest (Lee et al., 2018), increase accuracy over conventional image regency approaches. Deep learning (DL) algorithms, such as U-Net, provide additional enhancement of semantic segmentation for biomedical (Ronneberger et al., 2015) and plant images (Chang et al., 2020). The U-Net architecture is composed of encoder and decoder architecture (Figure 1E). The first half of the architecture contained the encoder or backbone and extracts features from an image with multiple levels. The second half of the architecture, the decoder, uses features from the previous step. For separating object and background information, advanced encoders gather additional features from images and achieve higher accuracies (Hoeser and Kuenzer, 2020; Zhang et al., 2020). Hence, for segmenting, there is room for improvement because U-Net performs well in different soil conditions.

In this study, we examined the reduced time intervals for predicting PA and estimate FW at different growth stages. This study follows four steps. First, we applied the combination of DL for plant image semantic segmentation for better PA and features for plant shape. Second, ML-based prediction models used the extracted plant features to predict the PA at the early and late pre-flowering stages with biological replication. Third, we established a relationship between FW using PA in a pre-flowering stage. Finally, we compared the predicted FW with PAs from various training models and harvested FW at 23 days after sowing (DAS).

## Materials and Methods

**FIGURE 2:**
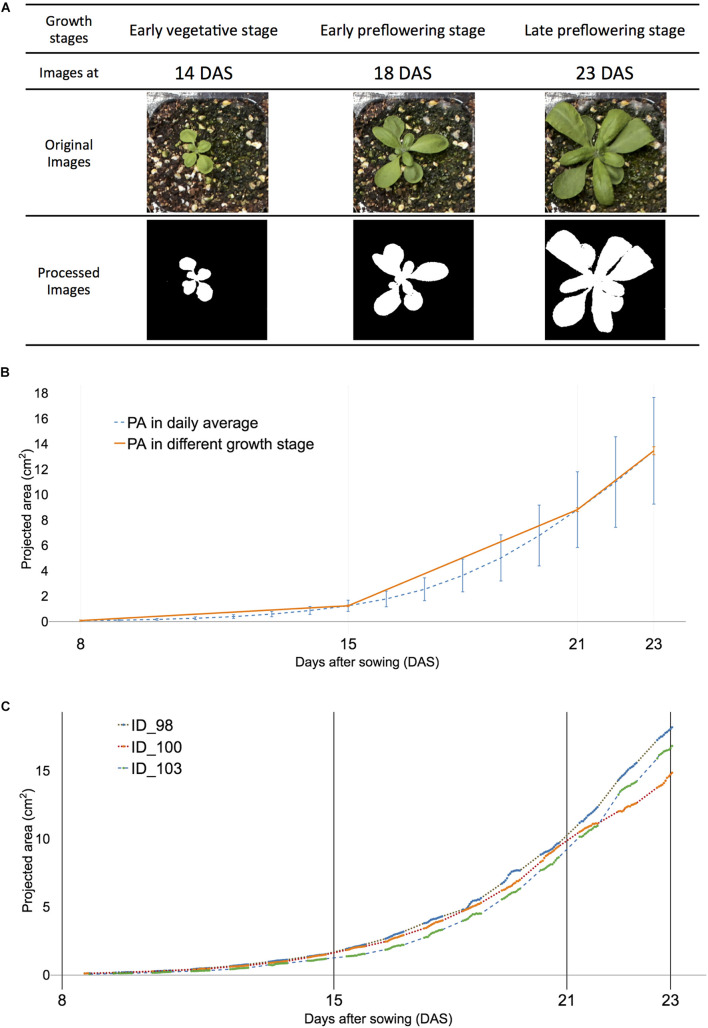
Definition of early vegetative, early, and late pre-flowering stages were used in the study to visualize corresponding projected area (PA) of all and selected samples of *Arabidopsis*. **(A)** Visualized plant images at three growth stages: early vegetative [8–15 days after sowing (DAS)], early pre-flowering stage (15–21 DAS), and late pre-flowering stage (21–23 DAS). **(B)** The visualized growth pattern of all samples. Dashed blue lines indicate the average PA in each DAS. The orange solid line indicates PA at early vegetative, early, and late pre-flowering stages, respectively. **(C)** The visualized growth pattern of selected individual Arabidopsis samples of ID 98 (dot), ID 100 (dash with lines), and ID103 (dash). The actual measurement time point is displayed with solid lines in each sample.

*Arabidopsis* developmental stages were defined as growth stages with early vegetative stage, early pre-flowering, and late pre-flowering stages from the phenological development of a plant (Boyes et al., 2001). The images of plants were acquired at all growth stages. However, the early pre-flowering stage was used for the late pre-flowering stage growth pattern (Figure 2A). We repeated experiment II to validate the outcome of experiment I at 23 DAS. We estimated fresh weight (FW) from PA with harvested plants at the early pre-flowering stage and compared the predicted FW with training models and measured FW at 23 DAS.

### Plant Materials and High-Throughput Phenotyping Setup

*Arabidopsis thaliana* was planted in the soil mixture and then moved to the HTPP with environmentally controlled conditions. The platform was programmed to obtain images with a 4K-RGB camera (Logitech, California, United States) every hour between 08:00 am and 7:00 pm during the photoperiod. A motorized irrigation dipper was connected to each tray and filled with water every 4 days over 4 weeks. Light-emitting diodes provided (Lumens, Seoul, South Korea) 16 h of lighting at 230 μmol/m^2^/s. A more detailed description is available in the study by Chang et al. (2020).

### Image Analysis Method and Evaluation of Semantic Segmentation

The image analysis pipeline was modified from the work of Chang et al. (2020) and comprised three parts. The first part was the pre-processing image step that detected edges of the tray (Figure 1B), corrected errors (Figure 1C), cropped, and saved individual plant images (Figure 1D). The second part was the segmentation process that tested U-Net (Figure 1E) with various encoders (Figure 1F) and selected a well-performed encoder (Figure 1G). The last part involved post-processing, which removed additional errors (Figure 1H) and extracted features from images (Figures 1I,J). This study tested multiple encoders using U-Net for more quality data from plant images.

Pre-processing of images was required for U-Net implementation. Firstly, we corrected image distortion of captured raw RGB tray images using four red markers in pre-processing; a tray image included 32- or 50-cell individual plants. Then, the corrected tray image was cropped for separating individual plants using the detected red marker coordinates (Chang et al., 2020 #74). The cropped images were properly scaled and padded for the U-net network size (512 × 512 dimensions). Secondly, the cropped, scaled, and padded RGB color image and mask image pairs were needed to train a semantic segmentation network; the mask image consisted of a black background and a white foreground (i.e., plant region). We selected an encoder such as Densenet, then performed training steps. Lastly, the cropped, scaled, and padded RGB color image inputs to the trained network, then only plant area was separated from backgrounds such as soil as an output (i.e., a mask image). Finally, the fully connected conditional random fields were applied to the segmented results for post-processing.

Cropped images were generated for image analysis, and 446 images were randomly selected to represent data for comparing different backbone approaches and source code available at Github (Yakubovskiy, 2019). Selected backbones are listed in Supplementary Table 2. To evaluate each backbone (encoder), data were randomly divided into two: 90 and 10% for training and data validation, respectively. Image augmentation such as flip, padding, blur, and sharpen using Python (Python software foundation, Beaverton, OR, United States) was performed to reinforce smaller training data (Buslaev et al., 2020). For each backbone, a total of 500 epochs of training was performed (Yakubovskiy, 2019). The trained model was evaluated using the validation dataset at the end of each epoch because an epoch has as many steps as training data.

Each model of the backbone was trained using binary dice and focal loss functions (Eqs 5, 7; we used beta value in Eq. 5). The dice and focal loss exhibited good performance for class imbalance problems (Milletari et al., 2016; Lin et al., 2017; Salehi et al., 2017; Zhu et al., 2019) [the class meant the foreground (plant part) and the background]. At the earlier stages of growth, the sizes of the plants were small. Therefore, the foreground class was much smaller, causing a class imbalance problem. To overcome this, we used a combination of loss functions during training.

The evaluation of the semantic segmentation used various methods such as the intersection-over-union (IoU) method (Yu et al., 2018). Eq. 1 shows that the IoU used calculates overlapped PA percentage using the intersection of the PA between the predicted (denoted by A) and ground-truth areas (denoted by B) over union PA between the predicted and ground-truth areas.

(1) $$\text{IoU}=\frac{\mathrm{Area}\,\left(A\cap B\right)}{\mathrm{Area}\,\left(A\cup B\right)}$$

F1-scores were used for evaluating semantic segmentation in agriculture (Bargoti and Underwood, 2017) and can be calculated from Eqs 2–4. From the precision calculation, a true positive (TP) result indicated that the output correctly predicted the pixels in PA, while a false positive (FP) result indicated that the output falsely predicted the pixels in non-PA. A TP and a false negative (FN) result indicated that the output failed to predict pixels in PA. Various backbones with U-Net could correctly determine PA if the IoU score was >0.5. A higher number indicated a more accurate prediction from the model. To compare the results, IoU and F1 scores were measured and calculated average values were used.

(2) $$\mathrm{Precision}\,\left(P\right)=\frac{\text{TP}}{\text{TP}+\text{FP}}$$

(3) $$\mathrm{Recall}\,\left(R\right)=\frac{\text{TP}}{\text{TP}+\text{FN}}$$

(4) $${\text{F}}_{\beta }\,\mathrm{score}=\left(1+\beta^{2}\right)\cdot \frac{\text{P}\cdot \text{R}}{\left(\beta^{2}\cdot P\right)+\text{R}},\beta =1$$

(5) $$\mathrm{Dice}\,\mathrm{loss}=1-{\text{F}}_{\beta }\,\text{score},\beta =1$$

(6) $${\text{p}}_{\text{t}}=\left\{ \begin{array}{c} p\,\mathrm{if}\,y=1\\ 1-p\,\mathrm{otherwise} \end{array}\right.$$

(7) $$\mathrm{Focal}\,\mathrm{loss}\,\left({\text{p}}_{\text{t}}\right)=-\alpha_{\text{t}}\cdot {\left(1-{\text{p}}_{\text{t}}\right)}^{\gamma }\cdot \log\left({\text{p}}_{\text{t}}\right),\alpha =0.25,\gamma =2$$

y ∈ {±1} means ground-truth class and p ∈ (0,1) is the estimated probability of the model for the class with label y = 1.

(8) Loss⁢function=Dice⁢loss+Focal⁢loss

### Time-Series Data Definition and Projected Area Prediction Models Construction

This study measured a PA at the complete growth cycle of 232 plant samples. Experiments I and II measured 122 and 110 samples, respectively. The growth cycle range is 10–23 DAS with 165 time steps that include 12-time steps per day. The time data format was in a sequential order ranging from 1 to 165 because multiple time points were not present with the DAS format. To express specific time points with DAS, the measured hours divided by 24 h were added after DAS. If images were taken 17 h at 23 DAS, the time point expressed as 23 DAS was (17/24 h). The training set was composed of a convex hull and compactness from extracted individual plant images.

Based on the phenological development of a plant, Boyes et al. (2001) defined *Arabidopsis* growth stages using the early vegetative stage, early and late pre-flowering stages with the Biologische Bundesanstalt, Bundessortenamt und CHemische Industrie (BBCH) scale. The growth stages of the early and late pre-flowering stages corresponded to 1.04 and 1.1 (Figure 2A) where the decimal point indicated the number of rosette leaves. The early vegetative stage was before 1.04. In our study, rosette leaves were manually counted for early and late flowering stages. The developmental stages and corresponding lengths of the early and late pre-flowering stages ranged from 15 to 21 and 21 to 24 DASs, respectively, and 60 to 140 and 141 to 165 time steps, respectively (Table 1), because inflorescence emerged at late 23 DAS in a partial plant population.

**TABLE 1:** Summary of reference points of each dataset with two-time scales used in the study.

**Training set**	**Reference timepoints**			
	**DAS with (Time steps)**			
	**Training period**		**Testing period**	
	**Start**	**End**	**Start**	**End**
Training 1	15 (60)	21 (140)	21 (141)	23 (165)
Training 2	16 (72)	21 (140)	21 (141)	23 (165)
Training 3	17 (84)	21 (140)	21 (141)	23 (165)
Training 4	18 (96)	21 (140)	21 (141)	23 (165)
Training 5	16 (72)	20 (127)	20 (128)	23 (165)
Training 6	17 (84)	20 (127)	20 (128)	23 (165)

*The time scale was recorded with the day format as days after sowing (DAS) with time steps. Moreover, the corresponding time steps are mention in the parenthesis next to the day format.*

The early pre-flowering stage was then divided into six training data sets, in those with endpoints at 20 and 21 DAS, respectively. The first four training sets were based on the training window: 15, 16, 17, and 18 DAS, with corresponding time lengths of 80, 68, 56, and 44 time steps, respectively (Table 1). Each of the training sets contained an ID, date, day, month, and experiment number. Figure 2C (orange solid line at ID 100) shows the plot of the measurement of PA of plants in the controlled environment in the daylight period only.

The last two training sets (5 and 6) were based on the training windows starting with 16 and 17 DASs with the corresponding time steps of 55 and 43, respectively. A summary of the reference time points for each set is listed in Table 1, where the entire experiment was termed experiment I. To verify the repeatability, an additional entire experiment, which was termed experiment II, was repeated.

**FIGURE 3:**
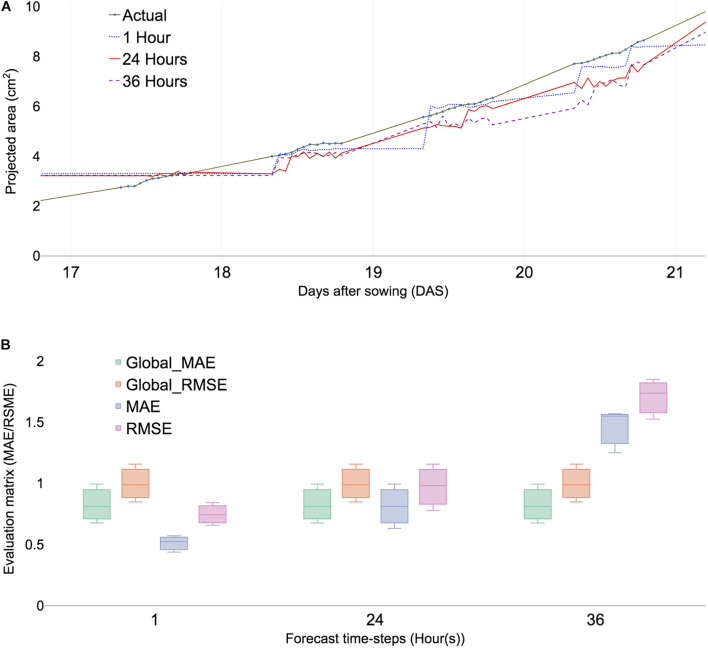
Predicted PA and error calculation at 1-, 24-, and 36-h forecast window sing ML algorithms (XGBoost). **(A)** Comparing PA prediction with 17–21 DAS of ID 98. **(B)** Comparing evaluation matrices of mean absolute errors (MAE), root mean square error (RMSE) of three selected samples, namely, ID 98,100, and 103. Global MAE and RMSE are defined as average MAEs and RMSE of all forecast time steps.

To examine the influence of various time lengths on the performance of the forecast model, a direct forecasting package called “forecastML” (Vienna, Austria) was utilized (Redell, 2020). The R forecast library required static (location) and non-static data (date and month). The period was set to 48 h. The overall scheme of the data structure is available (Supplementary Figure 3A). Individual model for each sample ID was constructed and evaluated as training 1 to 4 dataset (Supplementary Figure 3B) with multiple n-step ahead forecasting in training data hours, as shown in Figure 3A. The R code utilized in the analysis is available in the Supplementary Material. The mean absolute error (MAE) calculated the average errors using the sum of magnitude (absolute values) divided by the total samples (n), as shown in Eq. 9. The root means square error (RMSE) calculated average errors by identifying the total squared errors between the observed and the predicted values over n. The square root of mean squared errors was calculated using Eq. 10. The MAE and RMSE were the most used metrics for measuring the accuracy of time-series data (Cort and Kenji, 2005; Chai and Draxler, 2014).

(9) $$\text{MAE}=\frac{1}{\text{n}}\,\sum_{j=1}^{\text{n}}\left|{\text{y}}_{j}-{\hat{y}}_{j}\right|$$

(10) $$\text{RMSE}=\sqrt{\frac{1}{\text{n}}\,\sum_{j=1}^{\text{n}}{\left({\text{y}}_{j}-{\hat{y}}_{j}\right)}^{2}}$$

For all training datasets, horizons for the combined forecasting at 1, 6, 12, 24, 36, 42, and 48 h were selected.

### Trained Model Testing With Late Pre-flowering Stage Data

Individual PA forecasting models were constructed and tested for the late growth stage of the *Arabidopsis* plant. Four training models for various time intervals were then tested using testing sets ranging from 21 to 23 DAS (141–165 time steps), while two training models were tested with testing sets with a range of 20–23 DAS (133 to 165 time steps). Table 1 lists the reference time points for each set.

### Statistical Analysis

Statistical tests were performed using R (R Core Team, 2019). Three analyses were performed to verify that the forecast values from the ensemble model provided accurate output. The late growth time steps at 165 (23 DAS with 16 h) were selected for statistical analysis because inflorescence emerged at 23 DAS. First, an ANOVA test was used to determine if one or more datasets were different. The observed outputs were compared with the predicted values for six datasets. Non-significant datasets (*P* < 0.01) were selected and the homogeneity of variance for these sets was compared using Tukey’s honestly significant difference at a family-wise confidence level of 95%. The correlations between observed and predicted values were tested using Spearman’s rank coefficient (*R*).

### Measurement and Estimated Fresh Weight

Previous studies indicated that strong relationships between FW and PA exist in *Arabidopsis* (Fahlgren et al., 2015). Moreover, it was infeasible to harvest all representative plant images of 220,000 (165 time steps × 115 plants × 12 times per day). Therefore, plants were randomly selected and harvested among 112 plants from HTPP at 14, 17, and 20 DAS, respectively. Furthermore, 30 of 110 plants were randomly selected in experiment II at early 23 DAS because inflorescence emerged. Each plant was harvested and measured using the precision scientific balance (Ohaus, New Jersey, United States).

The following steps were required for establishing a relationship between FW and PA. First, a regression model was established to verify the relationship between FW and PA at the early pre-flowering stage using the data at 14, 17, and 20 DAS, respectively. Second, the regression model for the early pre-flowering stage was used to verify the predictability estimate of FW from PA in experiment II at 23 DAS. Third, FW was estimated from PA training models. Finally, the measured and predicted FW at 23 DAS were compared.

## Results

### Evaluation of Semantic Segmentation

The image analysis pipeline “U-Net” used for DL algorithms yielded good results (Chang et al., 2020). However, minor errors were evident when the network distinguished moss from plant areas. Thus, this study incorporated a more flexible U-Net network with various backbones (encoders) from other published networks to improve the segmentation task (Jiang and Li, 2020). IoU scores predicted PA over true PA values and a score of 1 indicated a perfect match between the predicted and true values. F1 score calculated model accuracy by combining precision and recall output. Similarly, a score of 1 indicated the highest value for the evaluation. Results from the evaluation matrix showed a high association between the evaluation and validation data (Table 2), indicating that the up-to-date backbones such as the SE-ResNext101 exhibited a reduced error rate than VGG16. Furthermore, the residual module-based network such as ResNet154 provided a high-confidence F1-score of 0.9613. The distribution evaluation matrix was then visualized to determine whether the network architecture influenced that of the output. The results (Supplementary Figure 1) indicated that squeeze and excitation (SE) architecture provided the most accurate PA among all backbones. The total loss of each backbone showed the same result from the F1 score (Supplementary Figure 1C). These results indicated that U-Net with SE backbones could be used for segmentation in various crops.

**TABLE 2:** Summary of an evaluation matrix for semantic segmentation of U-Net using various deep learning backbones (encoder).

**Backbones**	**Model evaluation matrix**		**Validation matrix**	
	**IoU**	**F1-score**	**IoU**	**F1-score**
VGG16	0.9384	0.9682	0.9272	0.9626
VGG19	0.9464	0.9724	0.9301	0.9637
SEResNet152	0.9665	0.9824	0.9323	0.9639
SEResNeXt101	0.9684	0.9839	0.9324	0.9648
SENet154	0.9697	0.9846	0.9314	0.9643
ResNet154	0.9565	0.9777	0.9259	0.9613
ResNeXt101	0.9623	0.9808	0.9281	0.9614
MoblieNetV2	0.9518	0.9749	0.9250	0.9608
InceptionResNetV2	0.9640	0.9817	0.9308	0.9637
DenseNet201	0.9609	0.9801	0.9310	0.9642

*Two evaluation scores are shown in the table. Intersection-over-union (IoU) evaluation matrix and F1-score were calculated.*

### Growth Pattern Analysis

The dynamic growth patterns were observed in *Arabidopsis* day and night (Wiese et al., 2007; van Es et al., 2019) and demonstrated that daylight growth was responsible for 70% of growth activities (Wiese et al., 2007). The overall growth pattern of the selected plant showed a somewhat linear trend for multiple growth stages (Figure 2B) and agreed well with previous studies on *Arabidopsis* (van Es et al., 2019). Three of 122 plant samples were selected and the dynamic growth pattern of the individual plants was compared (Figure 2C). Individual samples had distinct patterns from (orange solid line) one another and although the unmeasured night period varied, ID 98 had the fastest-growing rate ahead of ID100 and 102. However, its absolute growth rate (AGR) was the slowest at 20 DAS (Supplementary Table 2). Furthermore, the AGR of sample ID 100 grew fastest in selected samples of ID 98, 100, and 102; however, ID 102 was the fastest in afternoon time points. These results reveal that a dynamic growth habit can be observed within a 6-h time window. Consequently, the n-step forecast time was determined using multiples of 6 h and translated into 0.5 days because a 12-h-window was measured for a day.

### Prediction of the Projected Area With Training Models

High-confidence data were obtained using the 165 time steps collected and an up-to-date DL network-based image analysis. The definition of Boyes et al. (2001) was adapted (Figure 2A) for defining the developmental stages of *Arabidopsis*. Results showed that the early vegetative stage had slight sample variations from the pre-flowering stage of the plant developmental phase (Boyes et al., 2001; Figure 2B). Thus, the period from the early pre-flowering stage was tested, and the prediction models were validated in the late pre-flowering stage. Time-series analysis required the predefined time steps for training and testing purposes. Algorithms only used information within the training window to build a model and predict future values in the pre-determined forecast window. Later, predicted values from the trained model were compared with measured PA with U-Net within testing data. Forecasting terminologies were used in the time series analysis because not all data have true values in future events such as weather forecasts. This study determined the training and testing windows for the plant developmental stages until flowering bud emerged at late 23 DAS. The forecasting window at 24 h indicated 2 days after 12 h defined 1 day in the dataset. Various forecasting windows were tested with baseline studies to compare predicted and true values at the end of the late pre-flowering stage.

Verifying the essential time for the prediction model, the total time data was divided into six training sets following the start and end dates of the training data. Training sets 1–4 and training sets 5 and 6 were selected based on the end date of 21 and 20 DAS, respectively. Similarly, they were selected from the start point from 15 to 18 and 16 to 17 DAS, respectively (Table 1).

The initial analysis was performed with time points ranging from 15 to 21 DAS. Forecasting multiple windows and combining models provided more reliable results; however, the selection of time steps depends on the dataset (Galicia et al., 2019). The errors of different combinations of time steps were calculated using multiple-error evaluation matrices. The forecast value showed a similar trend among the different forecast windows at 1, 6, 12, 24, 36, 42, and 48 h, respectively (Supplementary Figure 2), because growth variation was observed at least 6 h (Supplementary Table 2). PA prediction deviated with increased time intervals for forecast and forecasting window at 24 h provided an additional reliable prediction value than the 36 h window (Figure 3A). The result indicated that the optimal forecasting window ranged between 24 and 36 h (Figure 3A). The data structure of the study was performed for 12 h a day for measuring daylight growth, which corresponded to 36 h for 3 days. To summarize, forecast windows at 1, 6, 12, 24, 36, 42, and 48 h corresponded to 0.04, 0.5, 1, 2, 3, 3.5, and 4 days, respectively.

Time-series analysis used various result-testing tools such as MAE and RMSE. MAE is one of the most commonly used matrices for measuring the performance of forecast models. A smaller MAE indicated that the predicted values were closer to actual values (Tay and Cao, 2001). The effectiveness of time-series analysis with the ML model was checked using RMSE (Chen et al., 2006). Two absolute error evaluation matrices provided appropriate information because no negative values exist in the dataset. Selected subsamples from training models were compared with check time window selection and forecast evaluation within testing data. The MAE of each plant sample showed little differences in multiple forecast windows (Figure 3B) and the overall error rate called the global MAE was 0.25 (Supplementary Figure 5A). Moreover, the RMSE of selected samples showed little differences (Figure 3B). All samples of MAEs were calculated using a forecasting window that ranged from 0.5 to 2 and a global MAE given as 0.7 (Supplementary Figure 4B). The result indicated that it served as a baseline MAE for other datasets.

A total of six training sets were generated from the endpoints of 20 and 21 DAS (Table 1 and Supplementary Figure 5F). The training period of the training sets 5 and 6 started at 16 DAS (Supplementary Figure 5F). The MAE ranged from 0.5 to 1.7 with the mean of global MAE as 0.7 (Supplementary Figure 4). The prediction errors decreased slightly compared with the baseline training sets 1–4. Furthermore, the training sets 3 and 4 with training windows that started at 17 and 18 DAS, respectively, were compared to check their error rate decreased with a narrow time. The results from training sets 3 and 4 indicated a similar error rate with training sets 5 and 6 (Supplementary Figures 5B–D). The mean of MAE training sets 3 and 4 was 0.6 and 0.7, respectively (Supplementary Figures 5C,D), suggesting that limited intermediated time points for time-series analysis can be feasible for predicting late-stage growth patterns.

The endpoint of the training set 5 and 6 was shifted to the forecasting window at 20 DAS. Training set 5 started a time window at 16 DAS and the MAEs of 1, 6, 12, and 24 h n-step forecast showed similar ranges (0.5–1.5) compared with the training sets 1–4. In the same training set, MAE increase by >2 or more at 36 and 42 h of the forecast period. Finally, the training set 6 with a start date at 17 DAS exhibited increased MAEs over 3 at 36 and 42-h forecast.

Overall, MAEs were increased after 36 h (3 days) of forecasting windows among different training sets (Figure 3B and Supplementary Figure 5F). This result indicated that models would predict reliable PAs at the endpoint of late time point in the testing time steps (Supplementary Table 3).

### Testing Trained Models With Late Pre-flowering Stage Data

**FIGURE 4:**
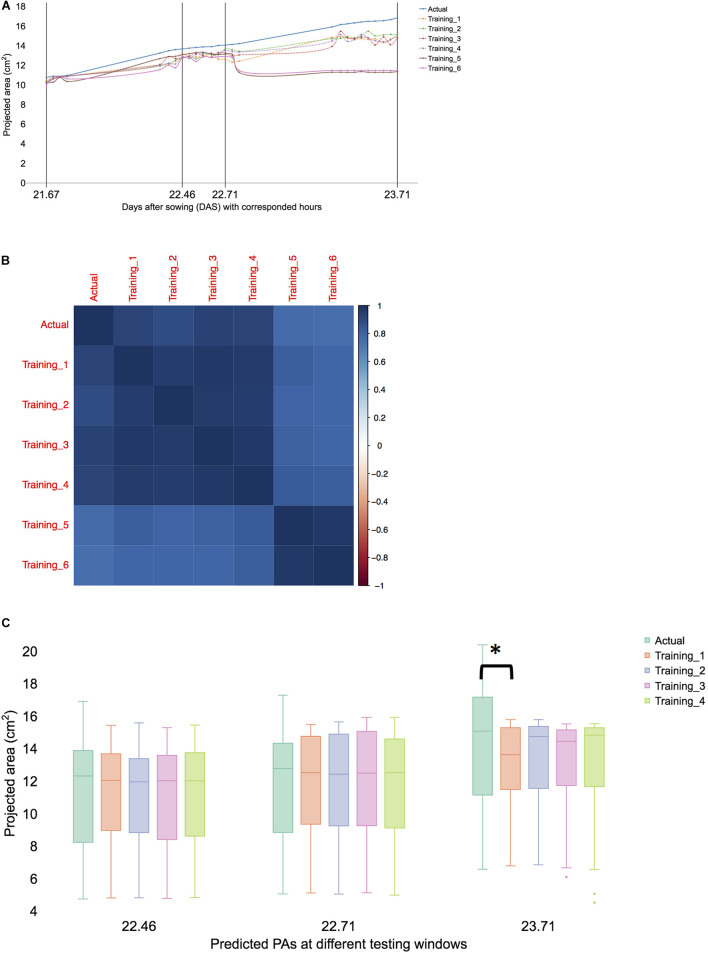
Predicted total leaf area and error range from 21 to 23 DAS in selected and total samples. Time points format as format images were taken at DAS (DAS.hours). MAE was calculated using multiple time windows with various forecast time points. **(A)** Predicted values with multiple time windows of the dataset (Figure 3) at 21–23 DAS (validation time points) of sample ID 98. **(B)** Correlation plot of all samples at 23.71. Clustering and grouping with R library for arranged samples. **(C)** Predicted PAs with training sets 1–4 at the selected testing window are given as 22.26, 22.71, and 23.71. The result is shown in a boxplot of actual and predicted PAs from each testing model. We compared with *post hoc* statistical test (Tukey’s HSD) and the significant result is showed with an asterisk.

This study forecasts the late pre-flowering stage using correlated features from the early pre-flowering. The growth forecast models for each training set (Table 1) were constructed and tested (Supplementary Figure 6 and Supplementary Table 3). The test time included the late growth stage of the *Arabidopsis* plant, including 23 DAS at which the emergence of inflorescence occurs in certain plants. To forecast the target days at 23 DAS, the training sets 5 and 6 were forecasted 42 h or longer. The forecasting plot of training sets 5 and 6 demonstrated that prediction at least 42 h ahead of time was feasible (Supplementary Figures 6E,F). Sample ID 98 was selected and all the predicted values of the six training sets were compared to evaluate prediction efficiencies (Figure 4A). Training sets 1–4 showed a stable trend in the whole growth period but training sets 5 and 6 demonstrated decreased accuracies after the end of 22 DAS. The prediction of the training sets 1–4 demonstrated close to actual values in the late growth stage at 23 DAS at 5:00 pm (Figure 4B). Moreover, the global error rate showed a similar trend in training sets 1–4 but different in training sets 5 and 6 (Figure 4C), although the error rates were similar between sets 1–4 and training sets 5 and 6 before 36 h of forecast window (Figure 4A). The result indicated that training sets 1–4 forecast the growth pattern of the late pre-flowering stage at 23 DAS. The training set 3 that included only 5 days of data showed similar MAEs compared with the 7-day data in the training set 1. An essential time window of fewer than 5 days of data (17–21 DAS) was generated, which included the transitional window from early to late pre-flowering stages in *Arabidopsis*. The same time window was tested in the replication at experiment II.

**FIGURE 5:**
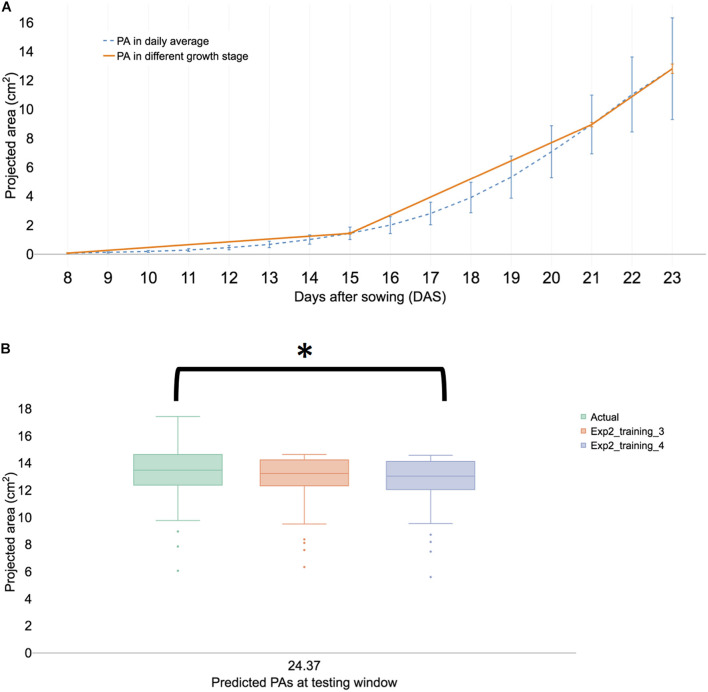
Summary of a biological replication study at experiment II. **(A)** The visualized growth pattern of all samples of biological replication experiment including three growth stages. **(B)** Predicted PAs with training sets 3 and 4 with testing window at 23 DAS. The result is shown in a boxplot of actual and predicted PAs from each testing model. We then compared with *post hoc* statistical test (Tukey’s HSD) and the significant result is shown with an asterisk.

In experiment II, the overall growth pattern was similar (Figure 5A) to experiment I (Figure 2B). HTTP stopped in the early hours of 23 DAS (23.37) because inflorescence was observed in the portion population (*n* = 110) and the 30 randomly selected plants for FW. Growth prediction models were constructed from 17 to 21 DAS (training set 3) and at 23 DAS, the *t*-test results of the observed and predicted values using the training dataset 3 were not different (*P* > 0.01). The Spearman’s rank coefficient (*R*) of PA and the predicted PA of experiments I and II were calculated and compared. The coefficient (*R*) of experiment I was 0.868 (Supplementary Figure 7A) and that of experiment II was 0.872 (Supplementary Figure 7B). The coefficient of each experiment was similar (*P* > 0.01), thereby confirming the experimental reproducibility. Furthermore, an additional statistical test is provided in Section “Statistical Analysis of Validation Sets.”

### Statistical Analysis of Validation Sets

The low MAEs (Supplementary Figure 5) is a good indication of high accuracy-ML models and provide statistically inseparable results with limited time points. To confirm the effectiveness of limited time for forecasting late growth stages, certain statistical methods were tested. One time point was selected in the late growth stage for statistical analysis. The selected time point was 23 DAS at 17 h (23.71) because this time point corresponds to the flower bud formation. The ANOVA test indicated that at least one training set was significantly different (*P* < 0.01). *T*-test of the observed and predicted values using training sets 1–4 were not significantly different (*P* > 0.01), while training sets 5 and 6 were observed to be significantly different (*P* < 0.01). Tukey’s honestly significant difference (HSD) test confirmed that all datasets of training sets 1–4 were not significantly different (*P* > 0.01) at 95% of family confidence level (Supplementary Figure 8). The result revealed that the time window ranges from 15 to 21 DAS were not different from the time-reduced windows from 17 to 21 DAS. In the experiment, I, the prediction of PA in the training 3 models was not significantly different from the actual PAs (*P* > 0.01) at 95% of family confidence level (Figure 5B and Supplementary Figure 9). Both experimental results confirm that 17–21 DASs was the essential time window for predicting at 23 DAS PAs. Furthermore, the selection of time intervals for HTPP was feasible because using a partial time interval was as effective as was using a whole interval in the early pre-flowering stage. This procedure might be applicable in detecting subtle differences in traits of interest where traits have expressed only a part of the life cycle of a plant. Moreover, focusing on a restricted time window of digital phenotyping data could alleviate the heavy burden of big-scale research projects because they require considerable resources to obtain new information during the entire life cycle of a plant.

### Estimated Fresh Weight Using the Projected Area

Fresh weight provided important information of interesting traits; however, the measuring data required the destruction of samples, and obtaining the corresponding time-series data was difficult.

**FIGURE 6:**
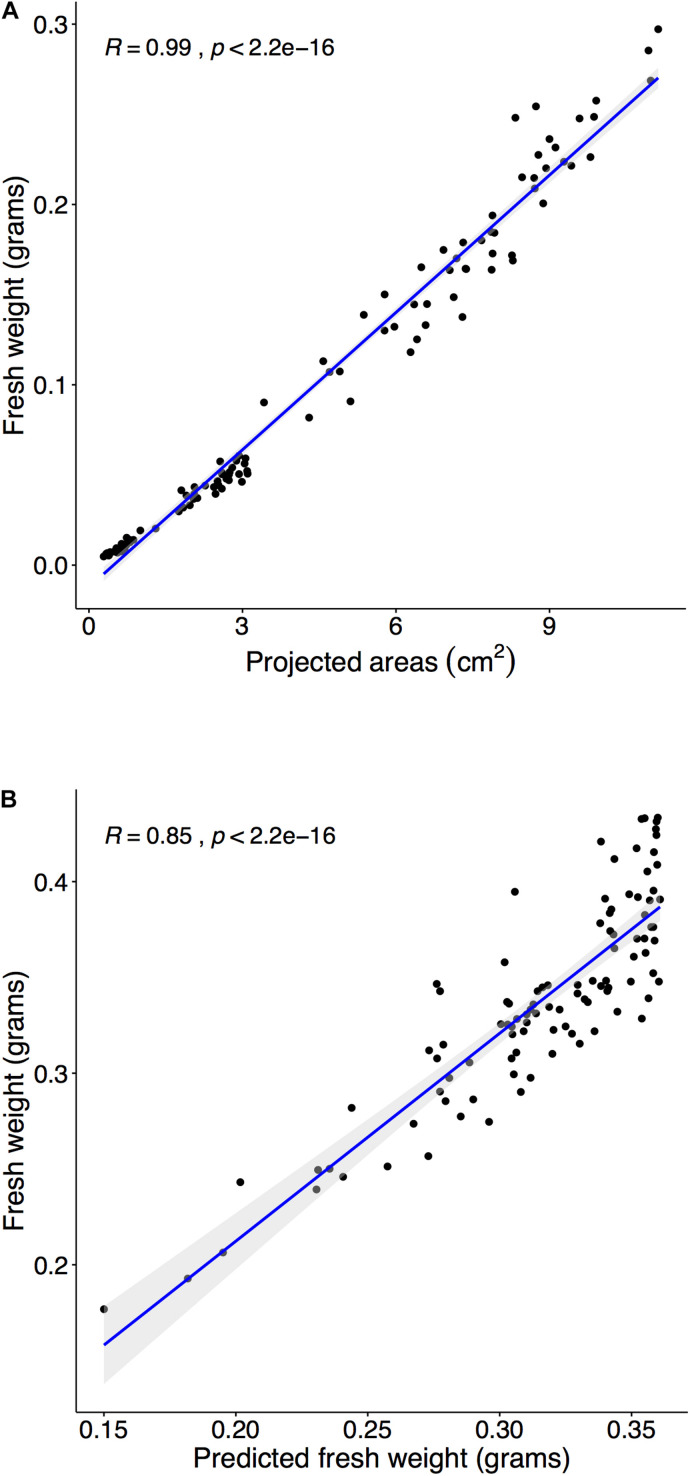
Estimating fresh weight (FW) from the PA and predict FW with three training sets. Panel **(A):** Harvest and weight 110 plants within the pre-flowering stage (15, 18, and 20 DAS), and we compared measured FW and PA from the image analysis. The correlation between FW and PA was tested using Spearman’s rank correlation coefficient (*R*). The coefficient’s (*R*) confidence level at 95% was shaded in gray. **(A)** Comparison of PA and FW in the range of 14–21 DAS (*R* = 0.9904). **(B)** Randomly select 30 samples at experiment II and predicted PAs with training 3 model and then converted into estimated FW. Compared estimated FW and measured FW at 23 DAS (*R* = 0.9042).

Multiple steps were required to predict FW using PA. The initial step was to establish a relationship between FW and PA in a target plant species. First, time-series data required corresponded to FW at each time point, and the estimated FW was obtained from the regression model between FW and PA in *Arabidopsis*. Previously, studies demonstrated a highly correlated relationship between FW and PA (Walter et al., 2007; Faragó et al., 2018), and the results of our study suggest the same relationship between FW and PA in the range of 14–20 DAS (Figure 6A). Moreover, the correlation coefficient between FW and PA was 0.99 (Figure 6A). The next step was testing the established relationship in different growth stages. The regression model from the early pre-flowering stage for FW (*R* = 0.9683) was constructed and was used to estimate the FW of the late pre-flowering stage. Results indicated (Supplementary Figure 10) a high correlation coefficient value (*R* = 0.9382) compared with the measurement during harvest at 23 DAS. Moreover, the estimated FW from PA not only provided accurate values in the same growth stage but can also be applied to different growth stages. The last step was to compare with measured FW and predicted PA from the training models. PAs were predicted with the training model 3 (Figure 5B) and then FW was estimated using the regression from the previous step. Finally, the predicted FW was compared with the measurement during harvest at 23 DAS, and a high correlation coefficient (*R* = 0.8512) was observed (Figure 6B).

In summary, the new strategy showed that limited growth period ranges were required to predict the growth pattern of different developmental stages. The predicted PAs were confirmed in an independent study and the PAs were converted into highly accurate FW values.

## Discussion

Transforming into digital agriculture requires various digital (Redmond Ramin et al., 2018b; Weersink et al., 2018; Klerkx et al., 2019) and image data, which are essential data. The primary reason why digital agriculture is essential is to detect the health and performance of plants in real-time in various environments. RGB images provide quantitative data in plant breeding and production (Araus and Cairns, 2014). The early image analysis from the legacy method or early application of ML (Pan and He, 2008) yielded partial success and not many plant scientists benefited from the quantitative data from the extract from RGB images. Many studies of plant segmentation were published and the result was difficult since the environmental conditions (lights, view of camera, soil conditions) were not identical in each experiment (Jiang and Li, 2020). ML- and DL-based image pipeline showed superb qualities over outdated legacy methods. The image analysis pipeline from ML showed promising results in its application to semantic segmentation in the rosette plant species named *Youngia denticulata* (Lee et al., 2018). U-Net was applied to separate irradiated and wild-type *Arabidopsis* plants (Chang et al., 2020). Additionally, botanists showed interest to apply up-to-date DL in HTPP data analysis (Jiang and Li, 2020). The encoder and decoder portions of U-Net (Figure 1E) showed several performances in various environments (Zhang et al., 2020). The encoder provides valuable information on whether various encoders at U-Net yield different results for interesting traits (PA) in agriculture (Jiang and Li, 2020). We demonstrated a more flexible way of applying networks to images for plant phenotyping (Figure 2). The SE network architecture demonstrated the highest confidence level among various backbones (Supplementary Figure 1). VGG-16, a simple network, provides high accuracy for the IoU score at 0.94, indicating a 94% of the images were correctly predicted with the combination of simple networks. Thus, applying and using image processing with DL still held certain challenges because of the lack of significant computing resources such as graphical process units or tensor process units. In a limited resource-research scheme, it should be beneficial to apply a simple network and gradually move to more complex network schemes. Importantly, it would be interesting to examine a specific encoder that could provide superior performance to detect the organs of plants such as flowers or other targets for interesting traits.

*Arabidopsis thaliana* was selected because it is a model plant for scientists and is rich in several noteworthy information. Moreover, the growth pattern of the gene function (van Es et al., 2019) and stress responses (Dhondt et al., 2014) was analyzed using time series.

To incorporate time-series analysis in an *Arabidopsis* research, many challenges in extracting and analyzing data from OMICS, including phenomics data associated with developmental stages were experienced. Previously, studies suggested that the growth pattern of long time steps provides valuable information on interesting traits (Dhondt et al., 2014); however, an additional investigation was not reported. Using complete time-series data is beneficial because dynamic growth habits were observed between 15 and 23 DAS (Figure 2C). Time-series data divided into developmental stages defined with the BBCH scale provided a more descriptive explanation (Boyes et al., 2001) and useful defined-data structure for analysis. To explain the end of the analysis, training and testing windows needed to be associated with developmental stages. Time-series analysis with XGBoost demonstrated better performance over other algorithms (Ji et al., 2019); however, this method was rarely used in biomass prediction or studying interesting traits. We applied multiple time steps with XGBoost and multiple correlated features to predict PA and the result was highly confident. A new analysis method that restricted time-series data within predefined developmental stages is helpful because relevant data on the relationship between or among different growth stages were accessible. Working with interesting traits with a full life cycle in a plant is time-consuming; therefore, it is possible to narrow down specific developmental stages using our method. Furthermore, our method can be used to reduce time intervals within the developmental stages. The method can be applied to predict traits of interest using HTPP data. Abiotic stress-related screening requires multiple resources because plants require testing over a long period. *Arabidopsis* plant showed stress effect after being exposed to the salt solution for 8 days (Geng et al., 2013). In the drought stress study, *Arabidopsis* demonstrated wilt symptoms after we stopped watering for 20 days. The total observation period of abiotic stress was ∼33 or 50% of the whole life cycle. A new method is beneficial to researchers who require to screen a larger number of samples using the HTTP because reduced time windows for a population provide extra time for screening another population.

The FW of a plant is an important selection criterion for bioenergy conversion using plants and other target materials. Since plant weight is obtained only after the growing plant is harvested or growth is completely stopped, understanding plant characteristics in a non-destructive method is a fundamentally essential research field in recent biology. Previous studies have demonstrated a positive correlation between FW and PA (Walter et al., 2007; Araus and Cairns, 2014; Faragó et al., 2018). Predicting FW from PA is plausible if there is a high correlation between two factors. A high correlation was found in our experiment and the estimated FW from PA using a different developmental stage was accurate (*R* = 0.93). The results indicated that the estimated FW in individual plants was possible from PA using time-series data and can be applied to predict FW or biomass in crops. FW of vegetable crops has essential information for the grower since FW of vegetables is a good indicator of yield at harvest. Vegetable crops are grown in vertical farming or controlled environment agriculture (CEA) and are important in food production and distribution, particularly during a virus outbreak where food movement is limited. Vertical farming produces more food in urban settings compared with field production (Benke and Tomkins, 2017). The estimation of FW using the visible spectrum is beneficial and should be incorporated into vertical farming. Though phenotype information, such as the leaf area index, has been used for plant status (Wang et al., 2017) in CEA, the estimated FW provides better plant status information and serves as a good yield indicator (Marondedze et al., 2018). In a plant factory setting, accurate yield prediction was performed with early time-series phenotyping data in lettuce (Nagano et al., 2019). We tested a model plant in the CEA for growth forecast with a limited time window and it yielded an accurate result (Figure 6B). A forecast of lettuce FW is possible but accurately predicting individual FW of lettuce is challenging because vertical farming production plants are tightly placed because the indoor farming space is limited (Redmond Ramin et al., 2018a). Advanced DL network using various encoders with U-Net provides more FW or PA-related features. Furthermore, more sophisticated DL for time-series analysis was promising in other fields, e.g., advanced DL-based network, long-short-time-memory (LSTM), or gate recurrent unit (GRU) outperformed the recurrent neural network (Khaki et al., 2020). A novel DL called temporal attention-based network (TCAN) can replace LSTM and GRU in certain tasks (Hoeser and Kuenzer, 2020; Jiang and Li, 2020; Yan et al., 2020). DL can achieve a performance level hitherto unachieved in conventional and ML algorithms. Gathering and analyzing using a long chain of time-series data enhances accuracy and increases the prediction window of FW with up-to-date DL.

In conventional agricultural research to date, the observation and selection of crops are possible only at a set time with the naked eye of breeders. Time-series data analysis from HTPP could provide valuable information. We applied up-to-date DL for semantic segmentation from HTPP data and analyzed selected pre-flowering developmental stages to forecast the growth pattern of the next growth stage in *Arabidopsis.* High-confidence F1-score (97%) was achieved using U-Net with SE-ResXt101 for semantic segmentation. This study reported that a part (17–21 DAS) of the developmental stages (*P* < 0.01) is sufficient for predicting the growth pattern of different developmental stages at 23 DAS. The result was confirmed with an independent study (*P* < 0.01). Moreover, FW prediction (*P* < 0.01) with HTPP time-series data is validated. The proposed method could be applied to forecast the growth or yield of leafy plants such as lettuce.

## Data Availability Statement

The original contributions presented in the study are included in the article/Supplementary Material, further inquiries can be directed to the corresponding author.

## Author Contributions

SC and J-BK designed the research. SC and MH performed the experiments and data analysis. SC and UL analyzed the data and wrote the manuscript. MH, YJ, and J-BK advised on the result and discussions. All the authors discussed the results and implications and commented on the manuscript.

## Conflict of Interest

The authors declare that the research was conducted in the absence of any commercial or financial relationships that could be construed as a potential conflict of interest.

## Publisher’s Note

All claims expressed in this article are solely those of the authors and do not necessarily represent those of their affiliated organizations, or those of the publisher, the editors and the reviewers. Any product that may be evaluated in this article, or claim that may be made by its manufacturer, is not guaranteed or endorsed by the publisher.
