# Supplementary material for: Time-Series Growth Prediction Model Based on U-Net and Machine Learning in Arabidopsis
Source: Front Plant Sci. 2021 Nov 11;12:721512. doi: 10.3389/fpls.2021.721512 (PMC8631871; doi:10.3389/fpls.2021.721512)
Supplement: Supplementary file 1 [file Data_Sheet_1.docx]

Supplementary Material

## Supplementary Figures


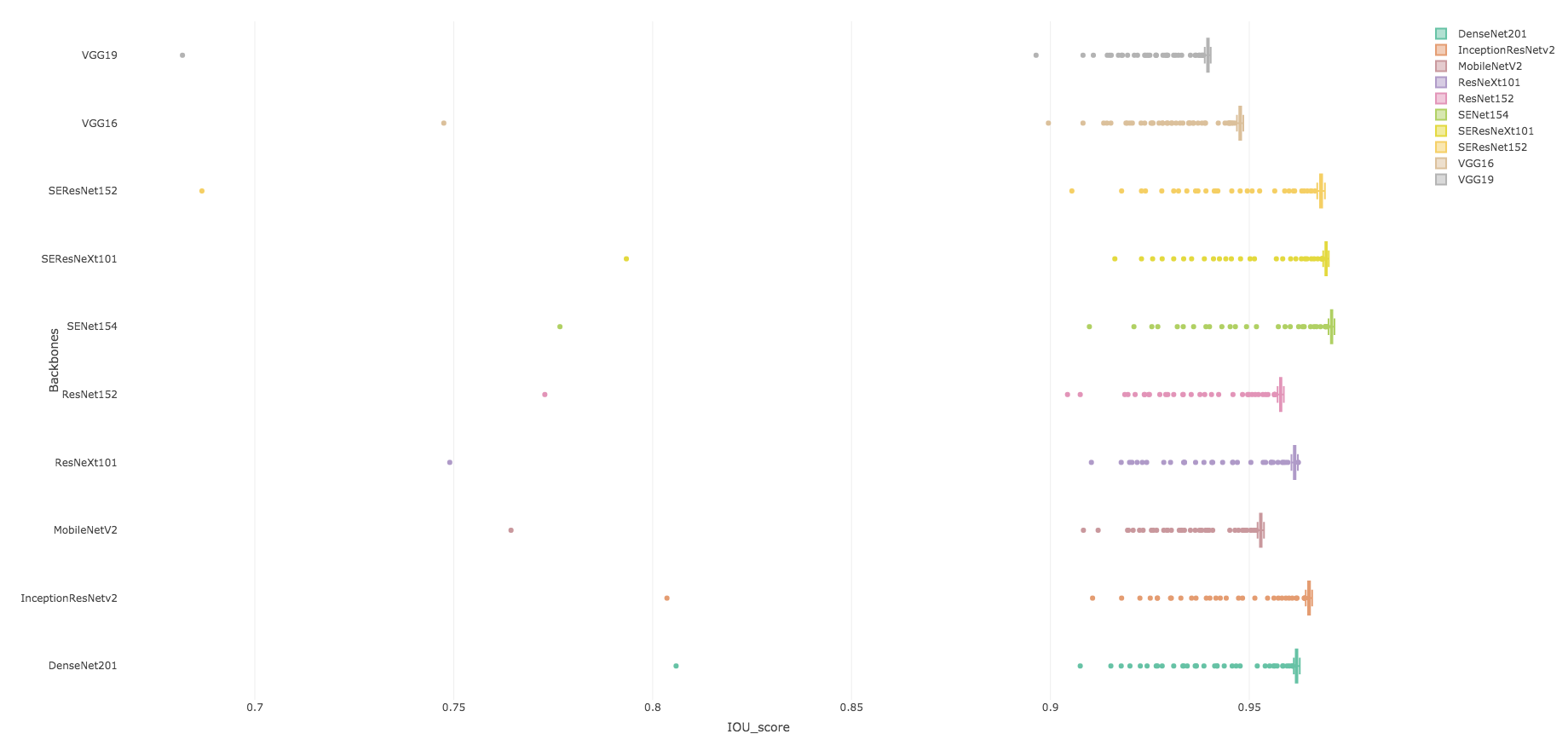


A


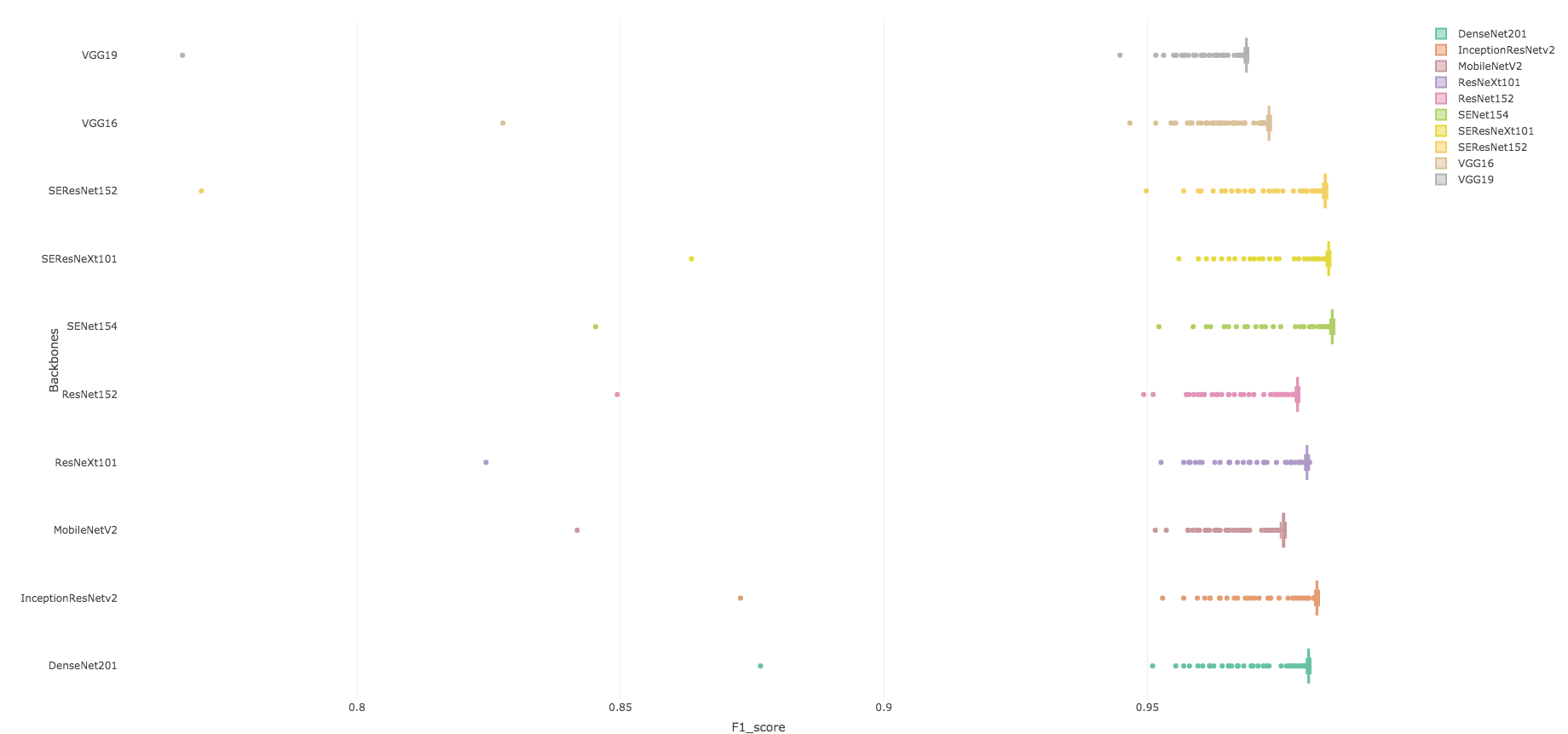


B


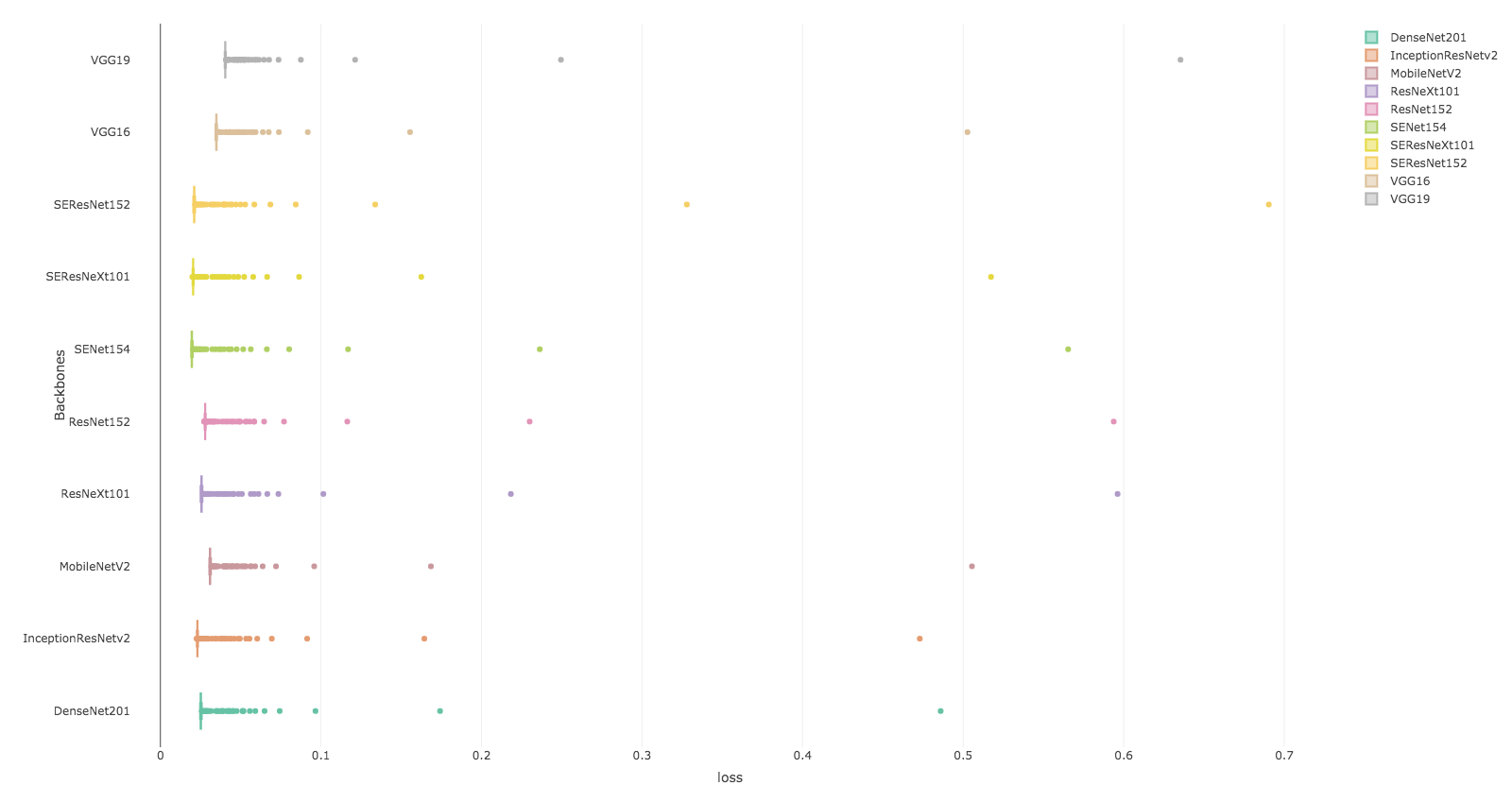


C

**FIGURE S1 |** Summary of evaluation matrix for semantic segmentation of U-Net with various deep learning backbones. (**A**) Box plot of each backbones with intersection-over-union (IOU) evaluation matrix. The X axis ranges were only shown from 0.7 to 0.99. (**B**) Box plot of each backbones with F1-score evaluation matrix. The X axis ranges were only shown from 0.75 to 0.99. (**C**) Box total loss of each backbones with loss function. The X axis ranges were only shown from 0.0 to 0.7.


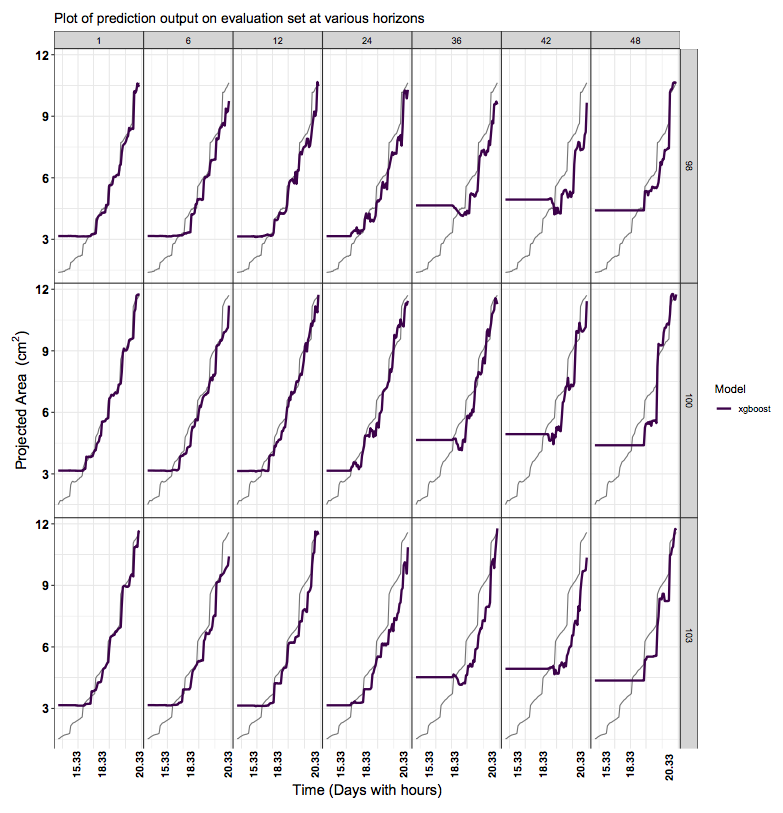


**FIGURE S2 |** Predicted total leaf area and error calculation from various horizons at 1, 6, 12, 24, 36, 42, 48 hour(s) with machine learning algorithms (XgBoost). ID number of 98, 100, and 103 samples were selected and visualized results. Each column indicated in various horizons and left side of row indicated the selected IDs for plant samples.

A


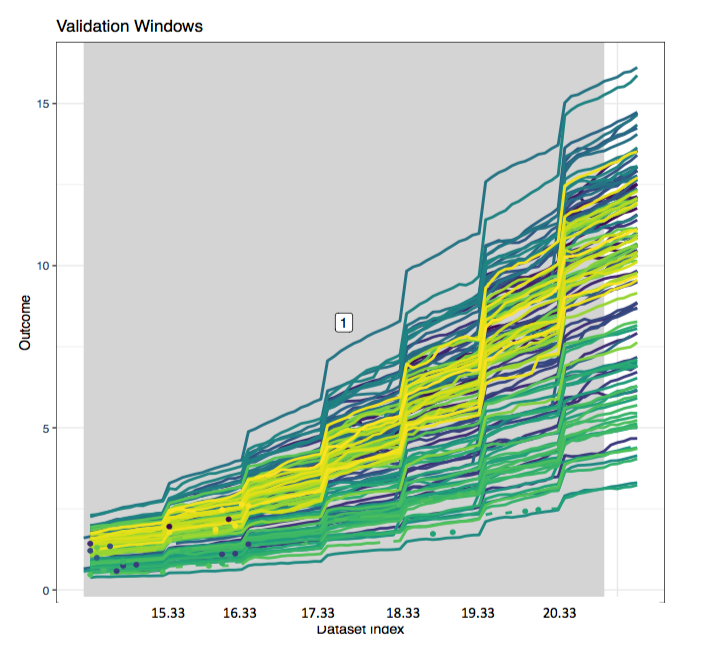


B

**FIGURE S3 |** Overall schematic of data and window size selection per each training set.

A

B

**FIGURE S4 |** Predicted total leaf area error calculation at 1, 6, 12, 24, 36, 42, 48 hour(s) with date ranges from 15 to 21 days after sowing (DAS). (**A**) Prediction errors at 1, 6, 12, 24, 36 , 42, 48 hours of sample ID 98,100, and 103. (**B**) Prediction errors at 1, 6, 12, 24, 36 , 42, 48 hours of all samples.

A

B

C

D

E


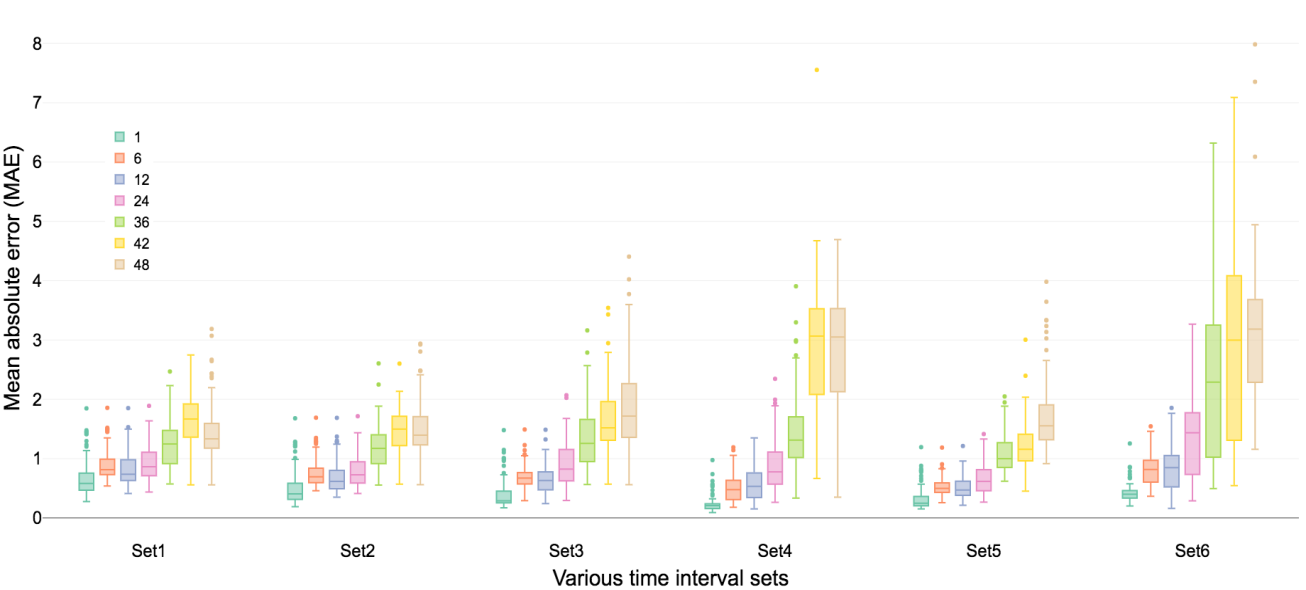


F

**FIGURE S5 |** Predicted total leaf area error calculation at 1, 6, 12, 24, 36, 42, 48 hour(s) with dates from various from 15 to 21 days after sowing (DAS). (**A**) Prediction of global errors with date ranges from 16 to 21 DAS. (**B**) Prediction of global errors with date ranges from 17 to 21 DAS. Panel (**C**) Prediction of global errors with date ranges from 18 to 21 DAS. (**D**) Prediction of global errors with date ranges from 16 to 20 DAS. (**E**) Prediction of global errors with date ranges from 17 to 20 DAS. (**F**) Prediction of global errors of all samples at the early preflowering stage.


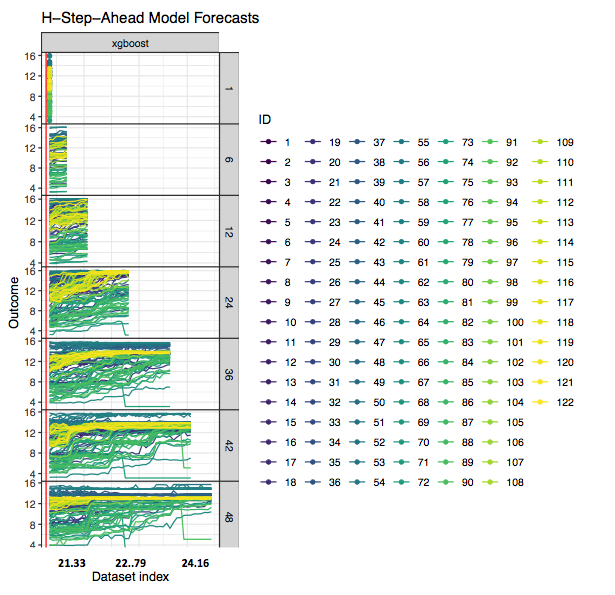


A


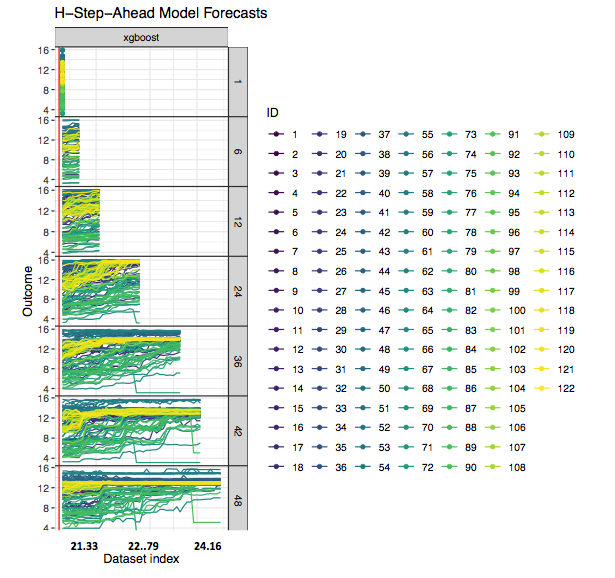


B


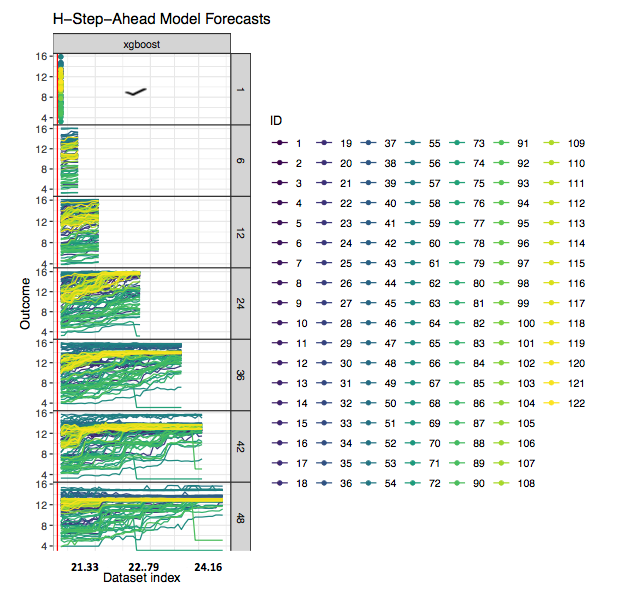


C


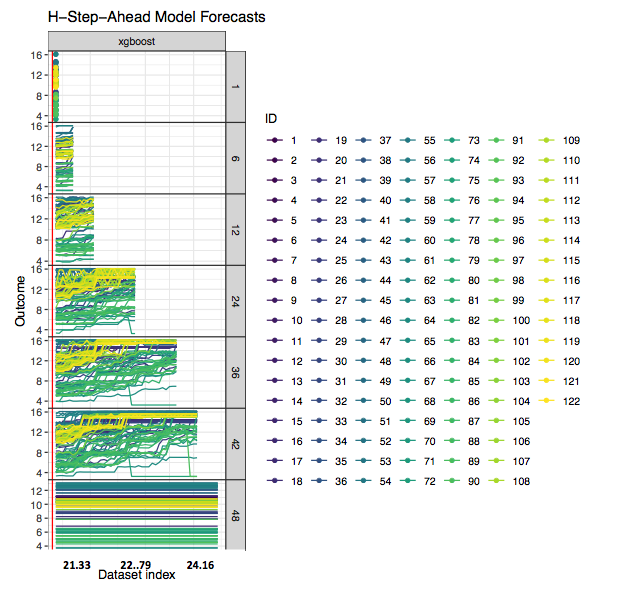


D


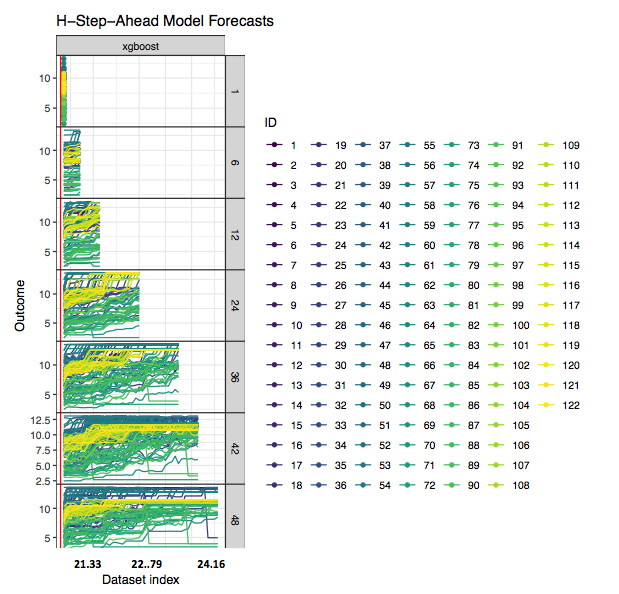


E


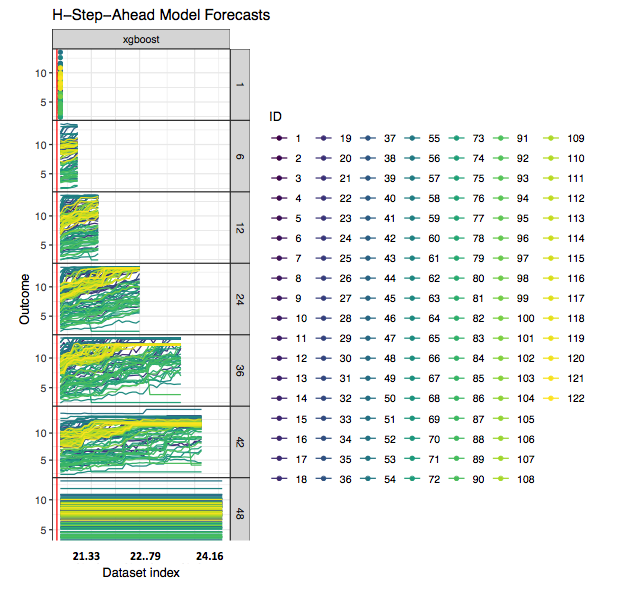


F

**FIGURE S6 |** Predicted total leaf area at 1, 6, 12, 24, 36, 42, 48 hour(s) with dates from 21 to 24 days after sowing (DAS). (**A**) Prediction values with trained model that date ranges from 15 to 21 DAS. (**B**) Prediction values with trained model that date ranges from 16 to 21 DAS. (**C**) Prediction values with trained model that date ranges from 17 to 21 DAS. (**D**) Prediction values with trained model that date ranges from 18 to 21 DAS. (**E**) Prediction values with trained model that date ranges from 15 to 20 DAS. Panel (**F**) Prediction values with trained model that date ranges from 16 to 20 DAS.

**FIGURE S7 |** Comparison of correlation coefficient (*R*) between the actual and predicted projected area (PA) of a plant between experimentsⅠand Ⅱ. TLAs at 23 DAS were predicted with 17–20 DAS growth prediction models. Correlation between the actual and predicted PA was tested using the Spearman’s rank correlation coefficient (*R*). The coefficient (*R*) confidence level at 95% was shaded in grey. (**A**) In the experiment Ⅰ, 120 samples were compared between the actual and predicted TLA (*R* = 0.868). (**B**) In the experiment Ⅱ, 110 samples were compared between the actual and predicted TLA (*R* = 0.872).

*

**FIGURE S8 |** Comparison of different training sets with the Tukey’s honestly significant difference (HSD) test at experiment Ⅰ. The family-wise confidence level at 95% was utilized. * Comparing projected area (PA) of training set 1 and training set 4.

**FIGURE S9 |** Comparison of different training sets with the Tukey’s honestly significant difference (HSD) test at experiment Ⅱ. The family-wise confidence level at 95% was utilized.

**FIGURE S10 |** Estimate fresh weight (FW) of sample at late preflowering stage (23DAS) projected area (PA) from established relationship between FW and PA in early preflowering stage (15 ~ 20 DAS). The correlation between FW and estimated FW was tested using the Spearman’s rank correlation coefficient (R). The coefficient (R) confidence level at 95% was shaded in grey (R = 0.9382).

## Supplementary Tables

**TABLE S1** |Selected lists of conversion of time series point to Posxcit format data.

| Time-steps | DAS | DAS.hours | Posxcit |
| --- | --- | --- | --- |
| 2 | 10 | 10.33 | 01/13/2020 |
| 60 | 14 | 14.79 | 3/12/2015 |
| 61 | 15 | 15.33 | 3/13/2015 |
| 72 | 15 | 15.79 | 3/24/2015 |
| 73 | 16 | 16.33 | 3/25/2015 |
| 84 | 16 | 16.79 | 4/5/2015 |
| 85 | 17 | 17.33 | 4/6/2015 |
| 96 | 17 | 17.79 | 4/17/2015 |
| 97 | 18 | 18.33 | 4/18/2015 |
| 108 | 18 | 18.79 | 4/29/2015 |
| 109 | 19 | 19.33 | 4/30/2015 |
| 120 | 19 | 19.79 | 5/11/2015 |
| 121 | 20 | 20.33 | 5/12/2015 |
| 132 | 20 | 20.79 | 5/23/2015 |
| 133 | 21 | 21.33 | 5/24/2015 |
| 144 | 21 | 21.79 | 6/4/2015 |
| 145 | 22 | 22.33 | 6/5/2015 |
| 156 | 22 | 22.79 | 6/16/2015 |
| 157 | 23 | 23.33 | 6/17/2015 |
| 165 | 23 | 23.79 | 6/25/2015 |

**TABLE S2** |Summary of absolute growth rate (AGR) in selected plant samples. AGR calculated with three different time intervals for comparing in each sample. Daily (D) time interval indicated ranges from 8:00 to 19:00, morning (AM) time interval indicated ranges from 8:00 to 13:00, and afternoon (PM) time interval indicated ranges from 14:00 to 19:00. Yellow shade indicated highest growth rate among samples and blue shade for medium growth rate and grey shade for lowest rate.

|  | Absolute growth rate (AGR) at different time intervals | | | | | | | | |
| --- | --- | --- | --- | --- | --- | --- | --- | --- | --- |
| Sample ID | 16 (D) | 16 (AM) | 16 (PM) | 20 (D) | 20 (AM) | 20 (PM) | 21 (D) | 21 (AM) | 21 (PM) |
| ID_98 | 0.516 | 0.281 | 0.795 | 0.829 | 0.280 | 0.477 | 1.144 | 0.528 | 0.544 |
| ID_100 | 0.470 | 0.249 | 0.185 | 1.131 | 0.603 | 0.489 | 0.626 | 0.348 | 0.211 |
| ID_103 | 0.352 | 0.229 | 0.098 | 0.936 | 0.339 | 0.523 | 0.802 | 0.374 | 0.332 |

**TABLE S3** |Predicted total leaf area and errors range from 21 to 24 days (testing) after sowing (DAS) in selected and total samples. Time points format as hours images were taken at DAS (DAS.hours). Mean absolute errors (MAE) were calculated from multiple time windows with various forecast time points. (**A**) Predicted values with multiple time windows of dataset (Fig. 3.) at 21 to 24 DAS (validation time points) of the sample 98. (**B**) MAE values from forecast at 1, 12, 24, 36, 42, 52 hours in 21 to 24 DAS in the sample 98.

| Dataset | Root mean square errors (RMSE) | | | | |
| --- | --- | --- | --- | --- | --- |
|  | 1 | 12 | 24 | 36 | 42 |
| Training_1 | 0.8879 | 0.9044 | 2.5131 | 4.5995 | NA |
| Training_2 | 0.9041 | 0.8705 | 2.3558 | 4.8753 | NA |
| Training_3 | 0.9901 | 0.7918 | 2.4746 | 5.1612 | NA |
| Training_4 | 0.7601 | 0.8111 | 2.4022 | 5.1784 | NA |
| Training_5 | 0.4996 | 0.6058 | 1.7408 | 4.6398 | 7.8512 |
| Training_6 | 0.1565 | 0.6367 | 1.7041 | 4.2496 | 14.6751 |

| Dataset | Mean Absolute Error (MAE) | | | | |
| --- | --- | --- | --- | --- | --- |
|  | 1 | 12 | 24 | 36 | 42 |
| Training_1 | 0.8094 | 0.6883 | 1.8622 | 3.7851 | NA |
| Training_2 | 0.8335 | 0.5318 | 1.7219 | 4.0554 | NA |
| Training_3 | 0.9191 | 0.5353 | 1.7919 | 4.3285 | NA |
| Training_4 | 0.6511 | 0.5568 | 1.7474 | 4.3011 | NA |
| Training_5 | 0.3123 | 0.4455 | 1.2569 | 3.8194 | 6.8246 |
| Training_6 | 0.0976 | 0.4558 | 1.2119 | 3.4163 | 14.1083 |

forecastML R code

setwd("/YOUR WORKING DIRECTORY/")

######forecastML implementation#######

library(forecastML)

library(dplyr)

library(DT)

library(ggplot2)

library(xgboost)

library(reshape)

######load raw data

Exp2_full =read.csv("Exp2_full_ts_test.csv")

Exp2_full = Exp2_full[,-1]

Exp2_full_test = subset(Exp2_full, date6 > 1)

######time step changes as posixct format

Exp2_full$newdate <- strptime(as.character(Exp2_full$date), "%y.%m.%d")

Exp2_full$newdate2=as.POSIXct(Exp2_full$newdate,format="%d/%m/%y",tz="GMT")

Exp2_full$month <- lubridate::month(Exp2_full$newdate2)

Exp2_full_test = subset(Exp2_full, date6 > 1)

#######data manipulation

Exp2_full_ts = Exp2_full[,c(10,2,3,4,5,6,7,8)]

str(Exp2_full_ts)

######renaming

Exp2_full_ts <- rename(Exp2_full_ts, c("date"= "newdate2"))

Exp2_full_ts <- rename(Exp2_full_ts, c("day"="date6"))

Exp2_full_ts <- rename(Exp2_full_ts, c("ID" = "ID2"))

str(Exp2_full_ts)

####

#write.csv(Exp2_full_ts,"Exp2_full_ts_test.csv")

########data split

##time 82,94,106,118,130,142

data = Exp2_full_ts

data = subset(data,day < 82)

data = subset(data,day < 146)

data_validation = subset(Exp2_full_ts,day > 140)

data_validation$exp <- as.numeric(factor(data_validation$exp))

data_validation$ID2 <- as.numeric(factor(data_validation$ID))

head(data_validation,3)

write.csv(data_validation,"data_validation.csv")

###

data = subset(Exp2_full_ts,day > 82)

data = subset(data,day < 146)

data = subset(data, compactness < 0.9)

data = subset(data, compactness > 0.1)

data$exp <- as.numeric(factor(data$exp))

data$ID <- as.numeric(factor(data$ID))

#######paramter setting & visualized trained data

outcome_col <- 1 # The column position of our 'Area..cm2.' outcome (after removing the 'date' column).

horizons <- c(1, 6, 12, 24, 36, 42, 48) # Forecast 1, 1:6, and 1:48 days into the future.

lookback <- c(1:48) # Features from 1 to 48 days in the past

dates <- data$date # Grouped time series forecasting requires dates.

data$date <- NULL # Dates, however, don't need to be in the input data.

frequency <- "1 day" # A string that works in base::seq(..., by = "frequency").

dynamic_features <- c("day","month") # Features that change through time but which will not be lagged.

groups <- "ID" # 1 forecast for each group or buoy.

static_features <- c("exp") # Features that do not change through time.

type <- "train" # Create a model-training dataset.

data_train <- forecastML::create_lagged_df(data, type = type, outcome_col = outcome_col,

horizons = horizons, lookback = lookback,

dates = dates, frequency = frequency,

dynamic_features = dynamic_features,

groups = groups, static_features = static_features,

use_future = FALSE)

DT::datatable(head(data_train$horizon_1), options = list(scrollX = TRUE))

###

p_train <- plot(data_train)

p_train <- p_train + geom_tile(NULL)

p_train

###window setting

windows <- forecastML::create_windows(data_train, window_length = 62, skip = 0,include_partial_window = FALSE)

p2 <- plot(windows, data_train) + theme(legend.position = "none")

p2

# model training

model_function <- function(data, outcome_col = 1) {

#data <- data[!is.na(data[, outcome_col]), ]

indices <- 1:nrow(data)

set.seed(224)

train_indices <- sample(1:nrow(data), ceiling(nrow(data) * .8), replace = FALSE)

test_indices <- indices[!(indices %in% train_indices)]

data_train <- xgboost::xgb.DMatrix(data = as.matrix(data[train_indices,

-(outcome_col), drop = FALSE]),

label = as.matrix(data[train_indices,

outcome_col, drop = FALSE]))

data_test <- xgboost::xgb.DMatrix(data = as.matrix(data[test_indices,

-(outcome_col), drop = FALSE]),

label = as.matrix(data[test_indices,

outcome_col, drop = FALSE]))

params <- list("objective" = "reg:linear")

watchlist <- list(train = data_train, test = data_test)

set.seed(224)

model <- xgboost::xgb.train(data = data_train, params = params,

max.depth = 10, nthread = 4, nrounds = 40,

metrics = "rmse", verbose = 0,

early_stopping_rounds = 5,

watchlist = watchlist)

return(model)

}

###model cross validation

model_results_cv <- forecastML::train_model(lagged_df = data_train,

windows = windows,

model_name = "xgboost",

model_function = model_function,

use_future = FALSE)

prediction_function <- function(model, data_features) {

x <- xgboost::xgb.DMatrix(data = as.matrix(data_features))

data_pred <- data.frame("y_pred" = predict(model, x))

return(data_pred)

}

###Error matrix calculation

data_error <- forecastML::return_error(data_pred_cv)

######forecast function

type <- "forecast" # Create a forecasting dataset for our predict() function.

data_forecast <- forecastML::create_lagged_df(data, type = type, outcome_col = outcome_col,

horizons = horizons, lookback = lookback,

dates = dates, frequency = frequency,

dynamic_features = dynamic_features,

groups = groups, static_features = static_features,

use_future = FALSE)

DT::datatable(head(data_forecast$horizon_1), options = list(scrollX = TRUE))

for (i in seq_along(data_forecast)) {

data_forecast[[i]]$day <- lubridate::mday(data_forecast[[i]]$index) # When dates are given, the 'index` is date-based.

data_forecast[[i]]$month <- lubridate::month(data_forecast[[i]]$index)

}

data_forecasts <- predict(model_results_cv, prediction_function = list(prediction_function), data = data_forecast)

plot(data_forecasts)

plot(data_forecasts)

plot(data_forecasts, facet = group ~ ., group_filter = "ID %in% c(98,100,103)")

windows <- forecastML::create_windows(data_train, window_length = 0)

p_full <- plot(windows, data_train) + theme(legend.position = "none")

p_full

###

model_results_no_cv <- forecastML::train_model(lagged_df = data_train,

windows = windows,

model_name = "xgboost",

model_function = model_function,

use_future = FALSE)

data_forecasts <- predict(model_results_no_cv, prediction_function = list(prediction_function), data = data_forecast)

write.csv(data_forecasts,"data_forecasts_18DAS.csv")

DT::datatable(head(data_forecast), options = list(scrollX = TRUE))

data_combined <- forecastML::combine_forecasts(data_forecasts)

write.csv(data_combined,"data_combineds_set1_3days.csv")

# Plot a background dataset of actuals using the most recent data.

data_actual <- data[dates >= as.Date("2015-05-1"), ]

actual_indices <- dates[dates >= as.Date("2015-05-1")]

window_start <- c(as.Date("2015-04-20"))

window_stop <- c(as.Date("2015-05-10"))

windows <- forecastML::create_windows(data_train, window_start = window_start, window_stop = window_stop)

# Plot all final forecasts plus historical data.

plot(data_combined, data_actual = data_actual, actual_indices = actual_indices)

plot(data_combined, data_actual = data_actual, actual_indices = actual_indices,

facet = group ~ ., group_filter = "ID %in% c(98,100,103)")
